# Supplementary material for: ACSL4 Directs Intramuscular Adipogenesis and Fatty Acid Composition in Pigs
Source: Animals (Basel). 2022 Jan 4;12(1):119. doi: 10.3390/ani12010119 (PMC8749670; doi:10.3390/ani12010119)

Full original blots used for Figure 2C. Each blot membrane was cut based on the standard band positions and then incubated with the appropriate antibodies.
The bands in the article are marked by red lines

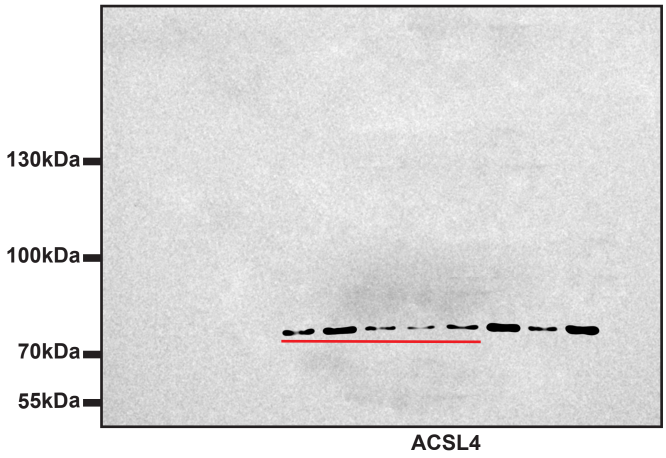

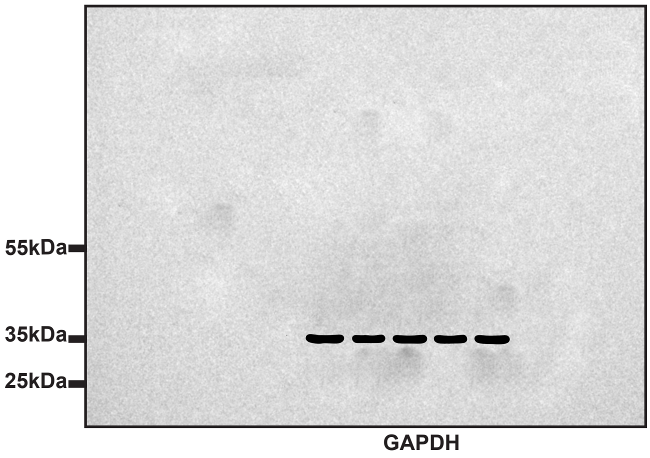

Full original blots used for Figure 3B. Each blot membrane was cut based on the standard band positions and then incubated with the appropriate antibodies.
The bands in the article are marked by red lines

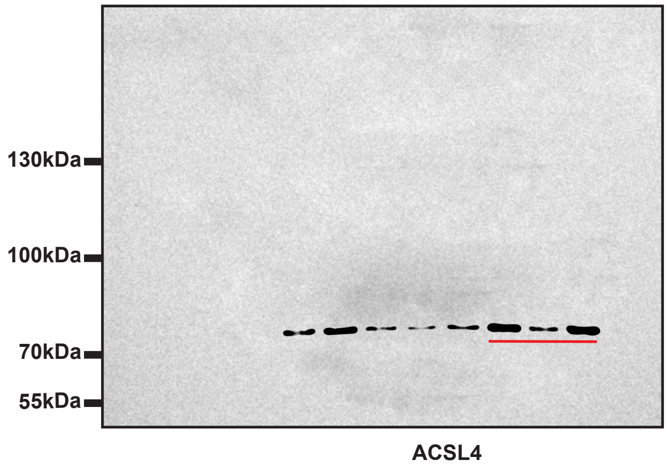

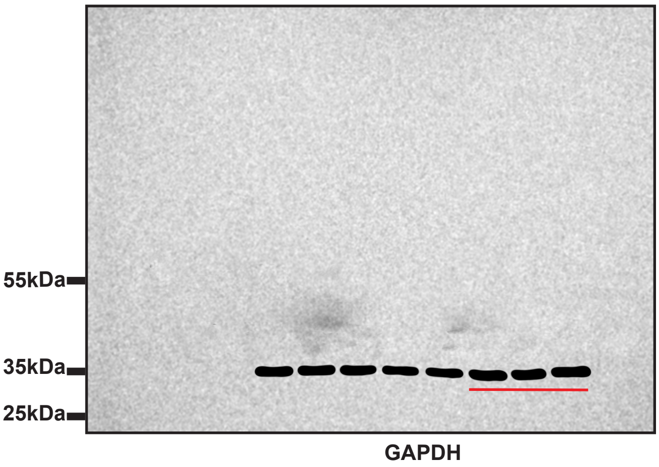

Full original blots used for Figure 4A. Each blot membrane was cut based on the standard band positions and then incubated with the appropriate antibodies.

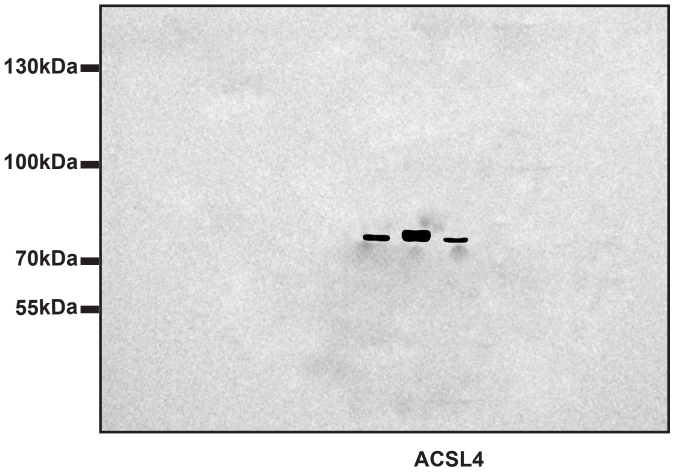

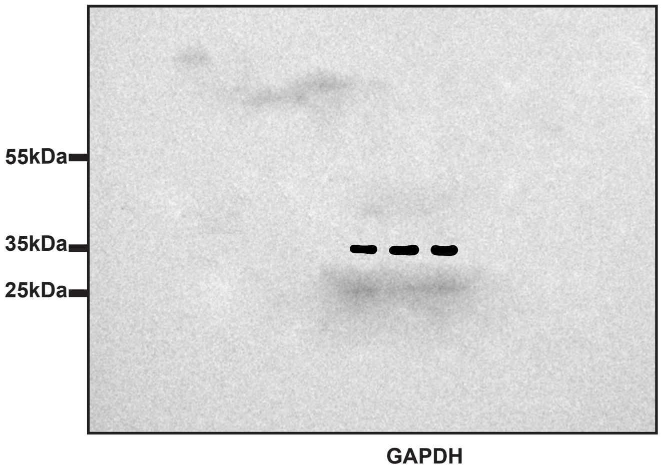

Supplement: Supplementary file 1 [file animals-12-00119-s001.zip › animals-1460961 - final - suppl/Figure S1. Western Original Image.pdf]
